# Exosomal microRNAs in diabetic heart disease

**DOI:** 10.1186/s12933-022-01544-2

**Published:** 2022-07-01

**Authors:** Dhananjie Chandrasekera, Rajesh Katare

**Affiliations:** Department of Physiology, School of Biomedical Sciences, HeartOtago, University of Otago, 270, Great King Street, Dunedin, New Zealand

**Keywords:** Diabetic heart disease, Exosomes, microRNAs, Biomarkers, Angiogenesis, Atherosclerosis, Ischemic heart disease

## Abstract

Diabetes is a metabolic disorder that affects millions of people worldwide. Diabetic heart disease (DHD) comprises coronary artery disease, heart failure, cardiac autonomic neuropathy, peripheral arterial disease, and diabetic cardiomyopathy. The onset and progression of DHD have been attributed to molecular alterations in response to hyperglycemia in diabetes. In this context, microRNAs (miRNAs) have been demonstrated to have a significant role in the development and progression of DHD. In addition to their effects on the host cells, miRNAs can be released into circulation after encapsulation within the exosomes. Exosomes are extracellular nanovesicles ranging from 30 to 180 nm in diameter secreted by all cell types. They carry diverse cargos that are altered in response to various conditions in their parent cells. Exosomal miRNAs have been extensively studied in recent years due to their role and therapeutic potential in DHD. This review will first provide an overview of exosomes, their biogenesis and function, followed by the role of exosomes in cardiovascular disease and then focuses on the known role of exosomes and associated miRNAs in DHD.

## Introduction

Type 2 diabetes mellitus is a chronic metabolic disorder that has a strong association with the development of cardiovascular diseases, referred to as diabetic heart disease (DHD) [1]. DHD is a broad definition encapsulating a cluster of conditions, including coronary artery disease, heart failure, cardiac autonomic neuropathy, peripheral arterial disease and diabetic cardiomyopathy [2–6]. A cohort study conducted by Shah et al. demonstrated that heart failure and peripheral arterial disease are the most common initial manifestations of cardiovascular disease in type 2 diabetic individuals [7]. Additionally, the Framingham study has shown that diabetic women had a higher risk of developing cardiovascular disease and congestive heart failure than diabetic males [4]. The Framingham study also implied the direct association between diabetes and the development of cardiomyopathy independent of any associated comorbidities [2].

Multiple studies have identified several molecular alterations in response to diabetes that underlie the development of diabetes-induced comorbidities [8–16]. Among these mechanisms, microRNAs (miRNAs) have been extensively studied. Results have shown a clear link between miRNA dysregulation and DHD [9, 10, 12–14, 17–21]. Of note, circulating miRNAs under DHD conditions have been demonstrated to have the potential to act as biomarkers [22–24]. Additionally, these circulating miRNAs can also affect the progression of disease under diabetic conditions[25–28]. Further, in addition to being circulated in naïve form, miRNAs can also be encapsulated into exosomes. Exosomes are a common nanovesicle that can encapsulate miRNAs and transport them in circulation [29, 30]. miRNAs isolated from plasma exosomes showed higher stability than miRNAs isolated directly from plasma [29]. Exosomes are efficiently engulfed by recipient cells, allowing miRNAs to exert their effects [31–33].This review will first provide an overview of the biogenesis and function of exosomes and miRNAs. Next it will focus on the role of exosomes in cardiovascular disease and the known role of exosomes and the miRNAs encapsulated within them in DHD.

## Exosomes

### Exosome biogenesis

**Fig. 1:**
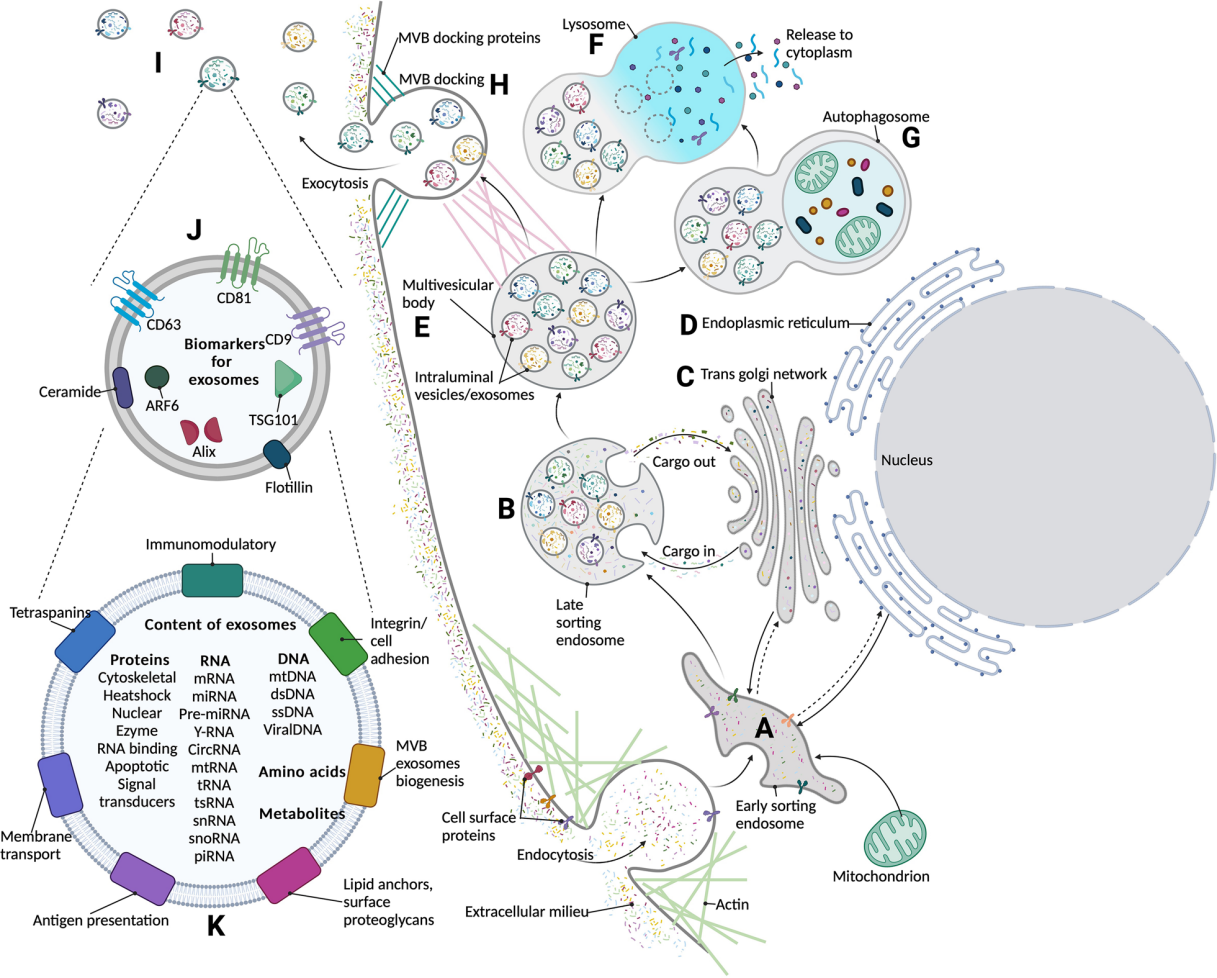
Exosome biogenesis. The invagination of the cell membrane results in the formation of the early endosome (**A**). Various budding into the endosomal lumen, initiates the process of forming exosomes/intraluminal vesicles in the late sorting endosome (**B**). Cargo is shuttled between the trans-golgi network (**C**), endoplasmic reticulum (**D**)and the late sorting endosome (**B**). Cumulatively, this leads to the formation of a multivesicular body (**E**) with fully formed intraluminal vesicles/exosomes in it. The multivesicular body can be further processed within the cell by the lysosome (**F**) or autophagosome (**G**) to breakdown the components of the multivesicular body into the cell. Otherwise, the multivesicular body can be docked (**H**) and fused to the cell membrane to release the exosomes (**I**) into the extracellular space. Exosomes can be characterized using different surface markers (**J**) that are consistently expressed by them. Exosomal functionality (**K**) is based on the varying cargo within the exosomes. Image Created with Biorender.com with valid license

Biogenesis of exosomes begins in the endosomal system, where the early endosomes mature into late endosomes [34]. During the maturation process, the endosomal membrane undergoes further invagination [34], resulting in the formation of intraluminal vesicles (ILVs) or exosomes within the organelles [34]. The accumulation of exosomes in the endosome results in the formation of multivesicular bodies (MVBs) [35]. The MVBs then fuse with the plasma membrane to release exosomes into the extracellular space [36]. The Ras associated binding protein (Rab) family of small GTPases is involved in intracellular vesicle transportation through cytoskeletal interactions and docking of the vesicles to the target compartments [36, 37]. The soluble N-ethylmaleimide-sensitive fusion attachment protein (SNAP) receptors (SNAREs) play a role in the docking and fusion of the MVB to the plasma membrane [38]. The exosomes released will have surface markers from their origin cells [39] (Summarized in Fig. 1).

### Exosome cargo sorting

The endosomal sorting complex required for transport (ESCRT) plays an essential role in forming ILVs in the endosome [40, 41]. Colombo et al. demonstrated that ESCRTs were involved in forming and secreting a heterogeneous subpopulation of exosomal vesicles [41]. They further showed that specific ESCRT components were involved in cargo loading in exosome biogenesis [41]. Other studies demonstrated an ESCRT-independent pathway of exosome biogenesis, as silencing of ESCRTs still resulted in the production of exosomes [37]. Tetraspanins such as CD63, CD9 and CD81 play an essential role in ESCRT-independent cargo loading into exosomes [42]. Tetraspanins organize the exosomal membrane into tetraspanin enriched domains [42, 43]. This formation is through the interaction between the exosome tetraspanins and other cellular tetraspanins, cytosolic proteins and lipids [42, 43]. Therefore sorting exosome cargos into exosomes occurs in both ESCRT-dependent and independent pathways [37, 44].

Interestingly, despite being a nanoparticle, exosomes can contain a variety of cargo. Studies have demonstrated the presence of various DNA molecules [45], RNAs such as miRNAs, circular RNAs and messenger RNAs [46], and proteins [47]. Multiple studies have also shown the functional importance of these cargos [32, 48, 49]. Exosome cargos are altered depending on the physiological or pathological conditions of the parent cells [49, 50]. Many studies have demonstrated selective chaperoning of miRNAs into exosomes under pathological conditions [51, 52]. As exosomes prolong the miRNA stability in circulation, they can also be considered a potential biomarker or a therapeutic target for various diseases [49, 50] (Summarized in Fig. 1).

### Exosome uptake

Exosome surface markers and cell surface markers determine the selectivity of exosome uptake by the recipient cells [48, 53, 54]. Exosomes can be taken up by nearby recipient cells (juxtracrine uptake), cells further away from donor cells (paracrine uptake), as well as by cells in distant tissues (endocrine) by travelling through the systemic circulation [23, 48, 55–59]. The uptake of exosomes by the selected host cells can occur through either the phagolysosomes, utilizing exosomal surface proteins, or phosphatidylserine receptors [48, 54, 55, 60]. Tian et al. demonstrated that exosomes were internalized through the endocytosis pathway, where the intracellular exosomes were trapped in vesicles and actively transported [53]. In contrast, Feng et al. demonstrated efficient exosome uptake by phagocytic cells, where the exosomes were targeted to large phagolysosomes [61]. They further concluded that the type of exosomal uptake was dependent on the type of cargos and surface markers expressed in the exosomes [61].

Once internalized, the exosomes release their cargo via the phagolysosome or by being localized in the late endosome [53, 61]. Tian et al. demonstrated that exosomal proteins separate from the exosomes approximately 3 h post-internalization, while exosome lipids were re-cycled into the plasma membrane [53]. Interestingly, Montecalvo et al. demonstrated that exosomes dock, bind and then fuse with the recipient cells, [62] thereby “injecting” the exosomal miRNAs into the recipient cells [62]. This injection allows the exosomal miRNAs to reach the recipient cell cytosol to initiate its functional processes [62]. Concerning the endocytosis exosomal uptake pathway, Tian et al. demonstrated that following endocytosis, exosomes were stored in lysosomes where the exosomal proteins were stored and utilized [55]. However, while they were able to determine successful miRNA transfer, they could not determine the exact pathway or mechanism of content release for exosomal RNAs [55] (Summarized in Fig. 1).

### miRNAs

miRNAs are short non-coding RNAs (~ 22 nucleotides) which post-transcriptionally alters protein expression by inhibiting the translation of messenger RNA. Under conditions such as diabetes, the alterations that occur at the gene coding regions lead to changes in miRNA expression [63, 64]. These changes induce pathophysiological alterations such as accelerated cardiomyocyte death, vascular remodelling, fibrosis, arrhythmias and stem cells dysfunction [8, 9, 12, 65, 66], all of which play a critical role in the progression of DHD [13, 67–69].

### miRNA biogenesis

The biogenesis of of miRNAs is initiated in the nucleus where the introns of protein-coding genes or independent genes are initially transcribed by RNA polymerase II/III to form primary miRNA transcripts (pri-miRNA) [70, 71]. The RNase III protein Drosha then processes the pri-miRNA with its essential cofactor DiGeorge syndrome critical region 8 (DGCR8), a microprocessor complex subunit to generate ~ 70 nucleotides long precursor miRNAs (pre-miRNAs) [72].

In the presence of the Ran-GTP cofactor, Exportin-5 binds specifically to pre-miRNAs, facilitating nuclear export of the pre-miRNAs into the cytoplasm[73]. The final stages of miRNA processing occur in the cytoplasm, where the pre-miRNA was then cleaved by the enzyme Dicer to generate the ~ 22 nucleotide miRNA duplex [70]. In order to generate mature miRNA, the miRNA duplex needs to be unwound [74]. Once unwound, the mature miRNAs are bound to argonaute proteins to maintain their stability within the cytoplasm [75].

### miRNA function

Mature miRNAs bind with RNA-induced silencing complexes (RISCs) to identify their messenger RNA (mRNA) targets[76, 77]. If the complementarity between the miRNA-RISC complex and the target mRNA is partial, the translation of the target mRNA leads to protein repression [78]. If there is exact complementarity between the complex and the target mRNA, the target mRNA undergoes cleavage and degradation [78, 79]. This feature of the miRNA allows it to target multiple genes and hence regulating multiple different pathological processes in its host cells [78–82].

### Physiological and pathological roles of miRNA

As miRNAs have a diverse range of targets, they control both physiological and pathological processes [83]. Physiologically, miRNAs regulate processes such as development, cardiac hypertrophy and neuronal function[83–85]. We along with others have demonstrated the crucial role of miRNAs in various physiological and pathological processes (*reviewed elsewhere*)[86–92]. For instance, studies have demonstrated that multiple different miRNAs, including miRNA-26b, -27a, -143 and -150, were upregulated in exercise-induced cardiac hypertrophy [93]. While another study showed significant modulation of miRNA-195, -125b, -199a, and -124 in response to pathological cardiac hypertrophy and heart failure [81], suggesting that different miRNAs respond to physiological or pathological hypertrophy. Furthermore, the cardiac-specific loss of miRNAs by deletion of DGCR8 results in the development of dilated cardiomyopathy and heart failure [85].

Studies have demonstrated alterations in plasma miRNA expression in response to type 2 diabetes [10, 12, 23]. In addition to their effects on host cells, miRNAs are also released into the circulation, affecting remote cells/tissues [94]. Once released into circulation, miRNAs are either bound to proteins, high-density lipoproteins or encapsulated in micro- or nanovesicles such as exosomes [94]. This encapsulation prevents miRNA degradation, increasing their stability in the circulation, allowing miRNAs to have various paracrine effects [29, 94]. Further, this stability also allows miRNAs to function as biomarkers for various conditions [50, 95, 96].

### Exosomes in CVD

#### Pathophysiological role of exosomes in CVD

**Fig. 2:**
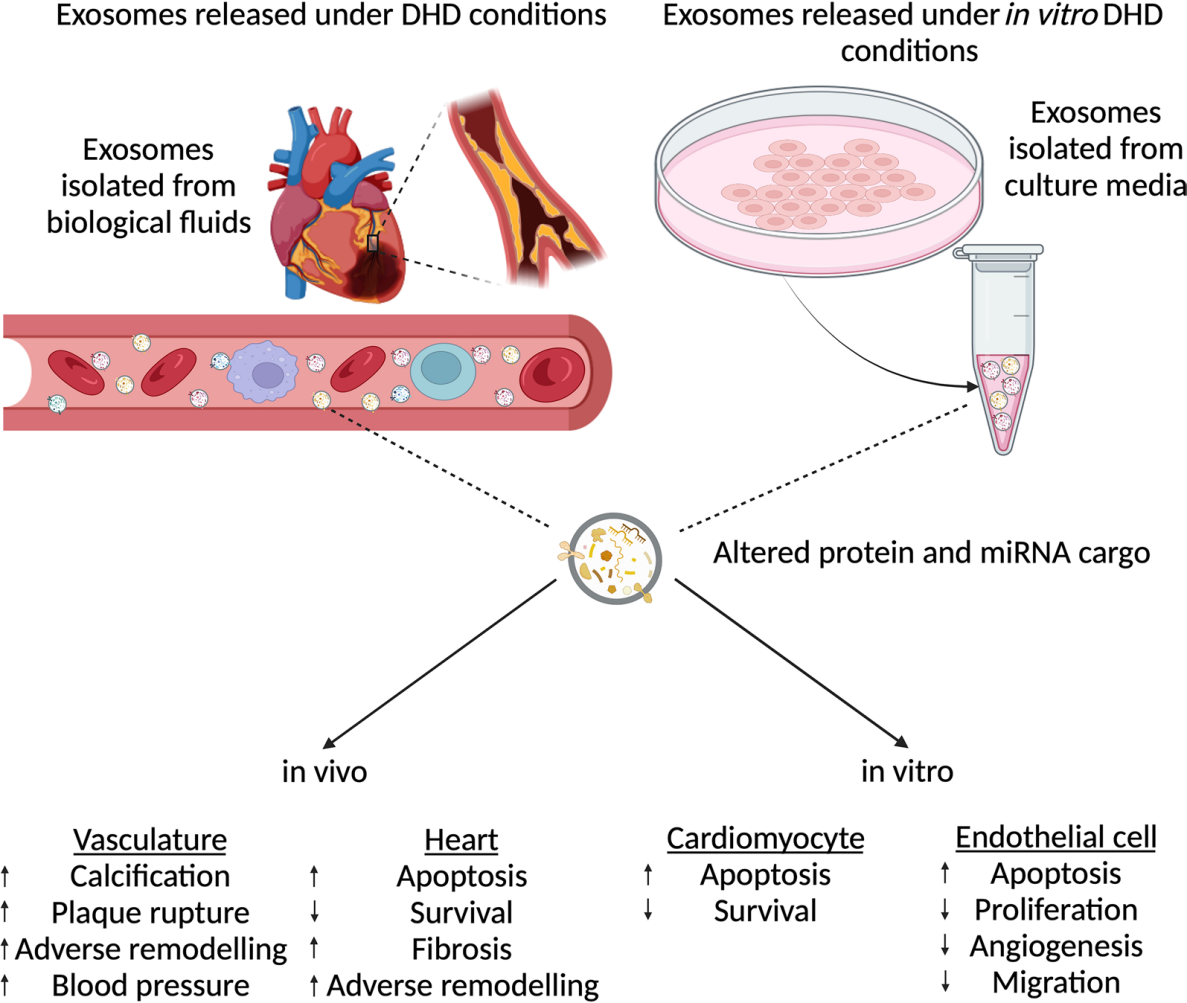
Function of exosomes under DHD conditions. Studies have been conducted on exosomes isolated from DHD biological fluids as well as from culture media under DHD conditions. Exosomes released from patients with DHD has been demonstrated to increase calcification, plaque rupture, adverse remodeling and blood pressure in the vasculature. This was mimicked in the in vitro experiments where diabetic exosomes increased endothelial cell apoptosis, while decreasing proliferation, angiogenesis and migration. Similarly, DHD exosomes in vivo resulted in increased apoptosis, fibrosis and adverse remodeling of the heart as well as reduced cell survival. Just as in in vitro experiments, DHD exosomes promoted cardiomyocyte apoptosis while reducing cardiomyocyte survival

Multiple studies have demonstrated exosomes to have various roles in CVD [31, 49, 57, 97–99]. Emmanueli et al. [23] demonstrated the potential of plasma exosomes as a biomarker in for recovery of patients post coronary artery bypass graft (CABG). They demonstrated increased density of the plasma exosomes post-CABG[23]. Importantly, these exosomes had increased expression levels of cardiac-specific miR-1, miR-24, miR-133a and miR-133b, both at 24- and 48-h post-surgery [23]. They also showed that positive correlation between alteration in the miRNA cargos within the exosomes and the expression of cardiac troponin I, a known biomarker for myocardial damage [23]. Another study demonstrated increased brain/head glycogen phosphorylase (PYGB) in circulating exosomes as an early and sensitive biomarker for cardiac injury [100]. The alteration of PYGB expression in exosomes isolated from rodents under doxorubicin-induced cardiac injury was observed before any alterations in the circulating cTn-I expressions [100], suggesting that in addition to miRNAs, exosomes can host protein cargos with biomarker potential. miRNA profiling of plasma exosomes from hypertensive rats showed exosomes could have a crucial role in hypertension [101]. Liu et al. demonstrated differential regulation of 27 exosomal miRNAs in spontaneously hypertensive rats compared to the control normotensive rats [101]. They attributed the differential miRNA expression to discriminatory packaging of the exosomal cargos under pathological conditions [101]. Further, another study demonstrated the role of circulating exosomal miRNA-194 that was upregulated in obesity to play a role in the development of cardiac injury and mitochondrial dysfunction [25]. Interestingly, treatment with a miRNA-194 sponge to downregulate the circulating miRNA levels attenuated obesity-related cardiac dysfunction in vivo [25] (Summarized in Fig. 2).

### Therapeutic potential of exosomes in CVD

**Table 1:** Pre-clinical studies showing the efficacy of exosomes in improving cardiovascular function

Source of exosomes	Type	miRNAs involved	Model	Effect	References
“Healthy” biological fluids	Pericardial fluids	let-7b-5p	In vitro	Improve cell viability Increase proliferation Increase networking capabilities	[49]
	Normotensive Wistar Kyoto rat plasms	–	In vivo	Decreased systolic blood pressure Reduced fibrosis Reversed hypertensive structural changes	[166]
	Heart tissue of exercised db/db mice	miRNA-29b miRNA-455	In vivo	Reduced cardiac MMP9 expression and function	[109]
Primary cardiomyocytes/CDC/CPC	CDC	miRNA-146a	In vitro	Promote angiogenesis Promote proliferation Decrease cell death	[104]
			In vivo	Promote angiogenesis Promote cardiac regeneration	
	Hypoxic CPC	miRNA-320, miRNA-222, miRNA-185	In vitro	Promote angiogenesis	[105]
			In vivo	Improve cardiac function Reduce fibrosis	
Endothelial cells/EPCs/Epithelial cells	EPC	miRNA-21a-5p miRNA-222-3p miRNA-221-3p miRNA-155-5p miRNA-29a-3p	In vivo	Reduced atherosclerotic plaques Ameliorate endothelium-dependent contractile dysfunction Reduce oxidative stress and inflammatory factors Improve vasodilation	[139]
	EPC	–	In vivo	Accelerate cutaneous wound healing	[177]
			In vitro	Promote migration Promote proliferation Promote tube formation Increase pro-angiogenic molecules	
	Endothelial cells	miRNA-126	In vivo	Improves neurological and cognitive function Increase axon density Increase myelin density Increase vascular density Increase arterial diameter	[26]
			In vitro	Increased primary cortical neuron axonal outgrowth Increases endothelial capillary tube formation	
Stem cells	human umbilical cord MSC	-	In vitro	Reduce cardiomyocyte apoptosis Promote tube formation Promote migration	[97]
			In vivo	Increase LV function Reduced fibrosis	
	human umbilical cord MSC	miRNA-19a	In vitro	Increased proliferation and migration Decreased apoptotic rate and proteins	[178]
			In vivo	Improved cardiac function post MI	
	hESC-pg	-	In vivo	Improve cardiac function	[107]
	MSC	-	In vivo	Ameliorate myocardial injury Reduce fibrosis and LV collagen	[120]
	MSC	miRNA-21a-5p	In vitro	Reduce pro-apoptotic gene products Reduced cel death in response to oxygen-glucose deprevation	[106]
			In vivo	Reduce pro-apoptotic gene products Reduce infarct size	
	bone marrow-derived macrophages	miRNA-146b miRNA-99a miRNA-378a	In vivo	Suppress inflammation Reduce necrotic lesion Reduce hematopoiesis	[138]
	induced pluripotent stem cells-derived MSC	–	In vivo	Enhance micro vessel density Improve blood perfusion in ischemic limb	[146]
			In vitro	Promote migration Promote proliferation Promote tube formation	
	bone marrow MSCs	miRNA-210-3p	In vitro	Promote migration Promote proliferation Promote tube formation	[147]
			In vivo	Increase blood perfusion Formation of new blood vessels	
	MenSCs	–	In vivo	Enhance neoangiogenisis Enhanced re-epitheliarisation	[102]
	Adipose-derived stem cell (ADSCs)	miRNA-30d-5p	In vivo	Decreased cerebral injury area Suppress autophagy Promote M2 microglia/macrophage polarization	[156]
			In vitro	Suppress autophagy	
	MSC	Let-7a miRNA-23a miRNA-125b	In vivo	Increase myelin thickness and axonal diameters of sciatic nerves Alleviate neurovascular dysfunction Improve functional recovery	[161]
Therapeutically modulated exosomes/ exosomes from therapeutically modulated source	Akt-overexpressing MSCs	–	In vitro	Promote angiogenesis Promote endothelial cell proliferation	[179]
			In vivo	Improve cardiac function Improve blood vessel formation	
	Transgenic (TG) mouse model with cardiac-specific overexpression of Hsp20	–	In vitro	Reduce apoptosis Improve angiogenesis	[99]
			In vivo	Promote exosome generation Reduce adverse remodeling Reduce apoptosis	
	Atorvastatin pre-treated bone marrow MSCs	miRNA-221-3p	In vivo	Facilitate wound healing Promote blood vessel formation	[149]
			In vitro	Promote migration Promote proliferation Promote tube formation	
	Antioxidant polyurethane nerve conduit with bone marrow stem cells derived exosomes	–	In vivo	Improve the neve functionality	[163]
	ADSC exosomes transfected with miRNA-93-5p mimic	miRNA-93-5p	In vivo	Reduce myocardial damage after acute MI Suppressed autophagy and inflammation after MI	[180]
			In vitro	Inhibit hypoxia-induced myocardial cell injury	

Exosomes have demonstrated both pro-and anti-angiogenic potential depending on the microenvironment [102, 103]. Using cardiosphere-derived cells (CDC), Ahmed et al. demonstrated that the CDCs treated with GW4869, a known exosome production blocker, lost their beneficial effects [104]. Results showed that CDC derived exosomes promoted angiogenesis and cardiac regeneration in scarred and infarcted hearts through activation of miRNA-146a [104]. This was supported by impaired protection against oxidative stress after injection of exosomes with miRNA-146a knockdown [104]. These data suggest that exosomes have therapeutic potential through multiple pro-angiogenic cargos.

Similarly, Gray et al. demonstrated an improved angiogenic potential of exosomes secreted by cardiac progenitor cells (CPC), extensively tested in CVD [105]. The exosomes produced by CPC under hypoxic conditions had increased angiogenic potential[105]. When added to the cultured endothelial cells, they increased the formation of networks compared to the exosomes produced from normoxic conditions[33]. Therefore, exosomes secreted under diseased conditions may aid in repair and regeneration [105]. In addition to angiogenesis, they also reduced the expression of profibrotic genes in rat fibroblasts that were stimulated with TGF-β [105]. Notably, treatment of rodent heart with hypoxic CPC exosomes significantly improved the cardiac function and reduced post-myocardial infarction (MI) fibrosis [105].

Exosomes have been studied for their effect on repairing the diseased heart through activation of the pro-survival signalling cascade. Zhao et al. demonstrated that intravenous administration of exosomes isolated from human umbilical cord mesenchymal stem cells (MSC) increased left ventricular systolic function and reduced fibrosis in a rodent model of MI [97]. This functional improvement was likely through inhibition of cardiomyocyte apoptosis, as in vitro studies showed that under hypoxic conditions, the exosomes increased the expression of anti-apoptotic protein Bcl-2 in recipient cardiomyocytes [97]. Luther et al. confirmed that MSC exosomes were enriched with cardioprotective miRNA-21a-5p [106]. Both in vitro and in vivo introduction of MSC exosomes could reduce pro-apoptotic gene products [106]. In vivo introduction of MSC exosomes to the mouse pericardial sacs 24 h prior to ischemic injury reduced infarct size [106]. The authors attributed the observed therapeutic effects of MSC exosomes to the presence of miRNA-21a-5p [106]. It would be interesting to determine the cardioprotective effects of exosomal miRNA-21a-5p under diabetic conditions.

Studies conducted by Kervadec et al. demonstrated that human embryonic stem cell-derived cardiovascular progenitors (hESC-pg) and the exosomes released by them enhanced the recovery of cardiac function in a rodent model of MI [107]. They showed an apparent alteration in cardiac gene expression in response to the hESC-pg and EV interventions [107]. In addition to their endogenic therapeutic potential, exosomes can be enriched by transfection with other pro-survival factors. Ma et al. enriched exosomes with pro-survival Akt (Akt-exosomes), which had a higher efficiency in improving cardiac function, and blood vessel formation in vivo under ischemic conditions [108].

Exosomes isolated from body fluid in patients with CVD have been used as a promising tool to understand the disease pathology and underlying molecular mechanisms. Beltrami et al. demonstrated increased expression of cardiac-specific miRNAs in exosomes isolated from human pericardial fluid of patients undergoing cardiac surgery compared to plasma exosomes [49]. Their study concluded that miRNAs originating from cells in the heart and its vessels, DICER, and AGO-2 (machinery involved in miRNA biogenesis), was encapsulated in the pericardial fluid exosomes [49]. They also demonstrated that treatment of endothelial cells with pericardial fluid exosomes under hypoxic conditions improved cell viability, proliferation, and networking capabilities [49]. Importantly, these effects were due to the miRNA cargo in the exosomes [49]. Chaturvedi et al. demonstrated that exercise increased the expression of miRNA-29b and − 455 in exosomes released by db/db mouse cardiomyocytes [109]. This increased miRNA expression was associated with a decrease in the Matrix metalloproteinase-9 (MMP9) protein [109]. As the downstream effects of MMP9 eventually lead to fibrosis and myocyte uncoupling, this study extrapolated that exosomes released during exercise could replace MMP9 inhibitors to manage cardiac remodelling [109] (Summarized in Table 1).

## Exosomes in DHD and their therapeutic potential

As previously mentioned, DHD is a broad definition comprises a cluster of conditions [2–6]. This section of the review will focus on the role of exosomes in the various comorbidities of DHD. Current literature on the exosome therapeutic potential in each comorbidity will be reviewed in this section.

### Effect of diabetes on exosomes

Although diabetes does not affect the size and concentration of exosomes, they have a significant effect in altering the exosomes cargoes, thereby affecting the functional ability of exosomes [31, 99, 110, 111]. Kim et al. attributed dysregulated glucose metabolism to the altered circulating exosomal miRNA profile in obese diabetic individuals in comparison to healthy controls [112]. Further, the crucial role for exosomes in diabetes is demonstrated in previous studies where transfer of plasma exosomes from obese mice induced glucose intolerance and insulin resistance, hallmark characteristics of type 2 diabetes, in lean mice [28]. This change in phenotype was achieved through the transfer of obesity related exosomal miRNA-192, -122, -27a-3p and 27b-3p that were significantly upregulated in the plasma exosomes of obese mice [28]. Similarly, adipose tissue macrophages (ATMs) exosomes of obese mice were able to induce insulin resistance and glucose intolerance in lean mice through the transfer of exosomal miRNA-155 [27]. Other studies have demonstrated the role of miRNA-26a in improving insulin sensitivity through circulating exosomes [113] and adipocyte derived exosomal miR-222 in inducing insulin resistance in hepatocytes and skeletal muscle by targeting insulin receptor substrate 1 [114].

Davidson et al. demonstrated a significant increase in cell death in response to exposure of exosomes isolated from cardiomyocytes that were exposed to hyperglycemia in order to mimic in vitro diabetic conditions [31]. In another study, intrinsic cell changes due to chronic hyperglycemia on heart explant-derived cells drove the production of exosomes which had impaired angiogenic and healing properties [115]. Similarly, exosomes isolated from primary cardiomyocytes cultures from Goto-Kakizaki (GK) type 2 diabetic rats, inhibited mouse cardiac endothelial cell (MCEC) migration, proliferation and tube formation in comparison to controls [110]. Increased expression of miR-320 in the diabetic exosomes was thought to be one of the underlying mechanism, as therapeutic knockdown of miR-320 restored angiogenesis in endothelial cells that were treated with diabetic exosomes [110]. Huang et al. demonstrated significant alterations in the transcriptome profiles of hyperglycemia induced stem/progenitor cell derived exosomes [111]. Chronic hyperglycemia has been demonstrated to reduce the cardiac expression of HSP20 [99]. HSP20 has been demonstrated to have cardioprotective effect as well as promoting exosome generation by direct interactions with the exosomal biogenesis pathways [99]. Interestingly, transgenic (TG) overexpression of HSP20 increased exosome generation which in turn improved cardiomyocyte survival and endothelial cell proliferation and migration under hyperglycaemic conditions [99]. These evidence confirms that alteration in the exosomal cargo plays a major role in deterioration of the exosomal functionalities under the diabetic condition (Therapeutic potential summarized in Table  1).

### Exosomes in diabetes-induced ischemic damage

Majority of the studies to date focused on either diabetes or ischemic heart disease (IHD), with only few of them studying the fate of exosomes in diabetes-induced heart disease including IHD. This is interesting as IHD has an increased association with diabetes, as the mortality rate due to MI is higher in diabetic individuals than the age and sex matched non-diabetic individuals [116] [117, 118], suggesting the need for more studies.

Davidson et al. studied the effects of exosomes isolated from diabetic patients undergoing coronary artery bypass graft surgery due to IHD [31]. In vitro cardiomyocytes treated with diabetic exosomes failed to reduce hypoxia-induced apoptotic cell death, which was attributed to the hyperglycemia-induced glycation of cardioprotective HSP70 in the diabetic exosomes. Further, they also demonstrated phosphorylation of cardioprotective ErK1/2 and Hsp27 by non-diabetic exosomes, but this was not observed in diabetic exosomes [31]. Liu et al. demonstrated increased expression of miR-144-3p in diabetic exosomes, which was associated with impaired ischemia-induced neovascularisation [119]. By downregulating v-ets erythroblastosis virus E26 oncogene homolog 1 (Ets1) in MSCs, miRNA-144-3p disturbed the MMP9 pathway, thereby disturbing the mobilisation of EPCs for neovascularisation [119]. Another study by Yuan Lin et al. showed that weekly intravenous injection of MSC derived exosomes in diabetic rats subjected to MI markedly reduced myocardial injury and fibrosis by inhibition of the TGFβ1/Smad2 signalling pathway [120]. They also showed marked reduction in the left ventricular collagen levels as well as improvement in lipid metabolism-related enzymes in the myocardial tissues [120].

Although limited, available evidence suggest that exosomes may be effective in the treatment of ischemic injury under diabetic conditions, warranting the need for further studies (Therapeutic potential summarized in Table 1).

### Exosomes in diabetic dyslipidemia

Dyslipidemia is commonly associated with type 2 diabetes. Dyslipidemia is primarily characterized by the increased levels of triglycerides and low density lipoproteins and decreased levels of high-density lipoproteins [121]. Studies have shown a linear association between obese dyslipidemia individuals and HbA1c levels in individuals with type 2 diabetes [122]. Ibrahim et al. demonstrated significant upregulation of serum exosomal miR-34a in type 1 diabetic individuals with dyslipidemia [123]. Further linear regression analysis demonstrated a strong independent association between exosomal miRNA-34a and total cholesterol, low-density lipoprotein and serum endoglin [123]. As this study was based on T1D [123], it will be interesting to determine if T2D have a similar expression profile. In another study, plasma exosome miRNA profiling showed a marked increase in exosomal miR-326 in type 2 diabetic individuals, a direct target of adiponectin receptor [124]. This was supported by inverse correlation between miR-326 and plasma adiponectin level [124]. While this study did not focus on dyslipidemia, it is well known that reduced adiponectin is associated with increased low density lipoprotein (LDL) and vLDL [125]. Further studies are required to determine the relationship between exosomes alteration and diabetic dyslipidemia.

### Exosomes in diabetes induced vascular diseases

Diabetes is associated with a two-fold increased risk of development of vascular diseases, independent of other conventional risk factors [126]. Once developed, these diseases further intensify the failing health of diabetic individuals unless immediately intervened [6, 7, 126–130]. This section will review the literatures that studied the modulation of exosomes in some of the common clinically relevant vascular diseases associated with diabetes.

### Atherosclerosis

Atherosclerosis is a disease in which abnormal metabolism or blood coagulation causes thickening and hardening of the arterial walls, resulting in narrowing and obstruction of the vascular lumen. Accelerated rates of atherosclerosis, hardening and narrowing of arteries along with the build-up of calcium phosphate salts leading to vascular calcification is commonly observed in diabetic individuals [2, 3, 131]. Vascular calcification predicts cardiovascular morbidity and mortality [132]. The degree of the vascular smooth muscle cells (VSMC) calcification was shown to affect the level of exosome release from the cell [131]. Further, elevated extracellular calcium resulted in the release of calcifying exosomes from VSMCs, leading to the further calcification of the cells [131]. It was proposed that the exosomal release pathway could, therefore, be a potential therapeutic target for the development of vascular calcification complications [131]. Other studies have demonstrated that aggregation of calcifying extracellular vesicles increases the formation of calcific mineral, leading to microcalcifications and ultimately, large calcification areas [133]. A dysregulated balance between apoptosis and the proliferation of VSMCs play a crucial role in intima-media thickening in individuals with diabetes [134]. Interestingly, Toliatto et al. demonstrated that endothelial cells derived exosomes (ECDE) disrupted the balance between antiapoptotic and pro-apoptotic signals in VSMC isolated from type 2 diabetic individuals thereby increasing its resistance to diabetes [134]. They also demonstrated that the diabetic ECDE had increased expression of membrane-bound platelet-derived growth factor-BB (mbPDGF-BB), which mediated the expression of miRNA-296-5p, an upstream regulator of the pro-apoptotic bak protein [134]. Exosomes are also demonstrated to play a role in plaque rupture. For instance, increased vasa vasorum angiogenesis in type 2 diabetes is demonstrated to promote the rupture of atherosclerotic plaques [135]. Interestingly, Wang et al. showed that insulin resistant adipocyte-derived exosomes promote vasa vasorum angiogenesis, suggesting another role of exosomes in diabetic atherosclerosis [135]. Li et al. demonstrated increased release of versican (VCAN)-rich exosomes by hyperglycemic HUVECs [136]. The uptake of the VCAN-exosomes by recipient VSMCs was associated with increased calcification and senescence leading to the progression of vascular damage [136]. Recently, Shyu et al. demonstrated that exosomes released by high glucose treated macrophages (hg-MExo) exhibited increased expression of MLAT1 by downregulation of its direct target of miRNA-150-5p as well as upregulation of the pro-atherosclerotic adipokine Resistin [137]. Furthermore, hgMExo treatment following arterial injury resulted in further increase in Resistin levels in the vascular tissues [137]. Interestingly, therapeutic downregulation of MLAT1 as well as upregulation of miRNA-150-5p resulted in a significant downregulation of Resistin [137].

In addition to the pathological role of exosomal alteration in the development of atherosclerosis, seminal evidence suggests that exosomes can be used to reduce diabetic atherosclerotic lesions. For instance, exosomes produced by bone marrow-derived macrophages, re-programmed inflammation and energy metabolism in recipient cells by transferring miRNAs that reduce TNFα and NF-κB, a useful therapeutic approach to treat atherosclerosis [138]. Bai et al. demonstrated that endothelial progenitor cell (EPC) derived exosomes was able to significantly reduce the progression of diabetic atherosclerotic plaques in a high fat diet fed C57BL/KsJ db/db mouse model [139]. This effect was independent of their effects on body weight and blood glucose levels [139]. Additionally EPC exosomes were able to reduce oxidative stress and inflammatory factors in circulation, as well as improving vasodilation in comparison to control treated groups [139] (Therapeutic potential summarized in Table 1).

### Peripheral artery disease (PAD)

Diabetes is an independent risk factors for PAD and the risk further increase with age [121]. PAD is generally defined by the partial or complete occlusion of either one or more peripheral arteries due to atherosclerosis [140]. Sorrentino et al. demonstrated that circulating exosomes from PAD patients increased VSMC migration, while inhibiting endothelial cells migration, thereby promoting adverse vascular remodelling [141]. Furthermore, they demonstrated distinct signatures of pro-inflammatory miRNAs in exosomes collected from patients with PAD [141]. One of the life-threatening complication of PAD in diabetic individuals is the development of chronic non-healing ulcers especially in the lower limbs, commonly referred to as diabetic foot ulcers (DFU) [142, 143]. Recent evidence suggests that exosomal miRNAs play a crucial role in regulating the process of DFU. RNA sequencing analysis of circulating exosomes showed marked upregulation of miR-20b-5p in patients with DFU. miR-20b-5p delayed wound healing by directly targeting the Wnt9b/β-catenin [144] and pro-angiogenic VEGF signalling pathway [145].

Several studies have tested the efficacy of exosomes in the treatment of PAD. Exosomes obtained from human induced pluripotent stem cell-derived MSCs, enhanced angiogenesis in mouse models of PAD [146]. Similarly, exosomes isolated from bone marrow MSCs were enriched with pro-angiogenic VEGF protein and miRNA-210-3p, which was able to induce formation of new blood vessels in an ischemic mouse limb model [147]. To date, there are no studies that have tested the effect of exosome treatment in diabetic PAD as a whole, although studies have demonstrated the therapeutic effects of exosomes in accelerating the healing of diabetic wounds. Li et al. demonstrated that exosomes derived from EPCs were able to promote re-endothelialization and promote healing of a cutaneous wound in diabetic rats [148]. Further in vitro studies showed that EPC exosomes were able to promote endothelial cell migration and proliferation as well as an increase the expression of pro-angiogenic molecules [148]. In another study Yu et al. demonstrated that exosomes derived from atorvastatin pre-treated MSCs was able to repair the diabetic wound by augmenting angiogenesis through activation of the AKT/eNOS pathway [149]. They also showed that atorvastatin pre-treated MSC exosomes were able to promote in vitro proliferation, migration, tube formation and VEGF levels in endothelial cells cultured under diabetic conditions [149]. The authors attributed the functional improvements were likely due to increased expression of miRNA-221-3p in the exosomes [149]. These findings were also supported by Dalifardouei et al. who showed the ability of menstrual blood MSC derived exosomes to promote healing of diabetic wound by promoting formation of new blood vessels through activation of pro-angiogenic VEGFA [102].

While the above studies have demonstrated the positive effects of exosome therapy in diabetic wound healing, the potential benefits of exosome therapy on other aspects of PAD are yet to be explored. However, due to the ability of exosomes to promote angiogenesis, it is likely, future research will be successful improving outcomes of PAD (Therapeutic potential summarized in Table 1).

### Stroke

Diabetes is an essential risk factor for ischemic stroke where a blood clot blocks or narrows the artery leading to the brain [150, 151]. Studies have shown that diabetes increases the risk as well as the severity of strokes [152]. Multiple studies have demonstrated that diabetes worsens patient outcomes following an ischemic stroke [150]. Expression of circulating exosomal miRNA-223 was associated with stroke occurrence, severity and short term outcomes, suggesting it as a potential biomarker [24]. Similarly, the serum exosomal brain-specific miRNA-9 and − 124 were considered as potential biomarkers in acute stroke [153]. Likewise, hyperglycemia related Fas and let-7b-5p in plasma exosomes was demonstrated to be a favourable prognostic biomarker for the prediction of poor neurological outcomes in ischemic stroke patients [154].

Interestingly, Venkat et al. showed that exosomes harvested from bone marrow stromal cells of type 2 diabetics were able to promote neuro-restoration and functional outcomes [155]. Further investigations showed that these exosomes reduced the expression of serum miRNA-9, which could be attributed to white matter remodelling and anti-inflammatory responses [155]. Studies conducted by Jiang et al. demonstrated that exosomes isolated from adipocytes derived stem cells enriched with miR-30d-5p were protective against acute ischemic stroke by suppressing autophagy and promoting macrophage polarisation [156]. Of note, increased autophagy is a consequence of chronic diabetes[8]. Following a stroke, T2D mice expressed significantly lower pro-angiogenic miRNA-126 in serum and brain tissues [26]. Interestingly, treatment with endothelial cells derived exosomes that express increased levels of miRNA-126, was able to promote neurorestorative effects in the post-ischemic T2D mice [26]. Furthermore, miRNA-126 was proven to enhance the therapeutic efficiency of EPC derived exosomes on diabetic ischemic stroke [157]. Additionally, the transfer of exosomes derived from bone marrow MSCs in to damaged neuron and astrocytes of diabetic mice was able to improve diabetes-induced cognitive impairment [158] (Therapeutic potential summarized in Table 1).

### Microvascular complications

Common microvascular complications in diabetes include diabetic retinopathy, neuropathy, and nephropathy. Research on exosomes associated with diabetes-induced microvascular complications is still in its infancy.

Huang et al. showed that increased levels of IgG laden exosomes in diabetic plasma could activate the classical complement pathway contributing to the development of diabetic retinopathy [159]. In another study, Gu et al. showed that under diabetic conditions, retinal pigment epithelial cells secrete miR-202-5p rich exosomes which prevented endothelial to mesenchymal transition, contributing to the pathological fibrosis in proliferative diabetic retinopathy [160].

Similarly, MSCs and Schwann cells derived exosomes alleviate peripheral neuropathy in diabetic models through the transfer of anti-inflammatory and pro-survival miRNAs [161, 162]. Recently, Singh et al. developed an antioxidant polyurethane nerve conduit consisting of nerve guidance channels incorporated with bone marrow stem cell-derived exosomes [163]. When implanted in an in vivo model of traumatic sciatic injury, the conduit with exosomes improved the neve functionality, suggesting this as a novel therapeutic approach for the treatment of diabetic nerve damage [163].

In a recent study, Wang et al. demonstrated that berberine attenuates podocyte injury caused by exosomes derived from high glucose-induced mesangial cells through TGFβ1-PI3K/AKT pathway, suggesting a role of exosomes in the development of diabetic nephropathy [164] (Therapeutic potential summarized in Table 1).

### Hypertension

There is a significant association between hypertension, high blood pressure, and the development of DHD [165]. Interestingly, aggressive blood pressure control in hypertensive and normotensive patients was demonstrated as an effective means to reduce the development of type 2 diabetes-induced complications [165]. Plasma exosomes miRNA profiling demonstrated that exosomes could play a crucial role in hypertension by regulating the TGF-β and MAPK signalling pathways [101].

Otani et al. conducted in vitro cross exposure experiments using plasma exosomes collected from spontaneously hypertensive rats (SHR) and Wistar Kyoto rats (WKR) [166]. They showed that treatment of WKR with plasma exosomes from SHR increased systolic blood pressure and induced structural changes like SHR phenotype. In contrast, treatment of SHR with plasma exosomes from WKR reversed hypertensive changes, including attenuation of structural changes in the left ventricles of SHR [166]. These results suggested that treatment with healthy exosomes may be of therapeutic value in hypertension. (Therapeutic potential summarized in Table 1) (Pathophysiological role summarized in Fig. 2).

### Diabetic cardiomyopathy (DCM)

DCM was first identified by Rubler et al. based on post-mortem observations of the cardiac autopsy from diabetic patients diagnosed with heart failure without other cardiovascular complications [167]. DCM has a 12% higher prevalence in type 2 diabetic patients than in healthy subjects [168]. It is a long-term and severe complication of sustained hyperglycemia that leads to increased cardiac oxidative stress, inflammation, abnormal Ca^2+^ handling and mitochondrial function, myocardial apoptosis and fibrosis [169]. Like with other components of DHD, only limited data is available to demonstrate the involvement of exosomes in DCM. Studies have shown that hyperglycemia can alter the exosomal cargo of diabetic cardiomyocytes, which can then transfer to the adjacent cells such as endothelial cells or fibroblasts to regulate their function [169]. For instance, Wang et al. showed that exosomes isolated from diabetic rat cardiomyocytes diminished the migration and proliferation of cardiac endothelial cells [110]. In contrast, exosomes isolated from non-diabetic rat cardiomyocytes promoted the migration and proliferation of cardiac endothelial cells [110]. They also showed marked upregulation of pro-apoptotic miR-320 and downregulation of angiogenic miR-126 and HSP20 in the diabetic exosomes [110]. In another study, Li et al. demonstrated enrichment of miRNA-320 in exosomes collected from individuals with type 2 diabetes. They proposed that miR-320 was e secreted by cardiomyocytes and taken up by the coronary endothelial cells, resulting in reduced nitric oxide production and inhibition of angiogenesis via decreases heat shock protein 20 [170].

Myocardial steatosis, accumulation of fatty infiltrated in the myocardium, is an independent predictor of diastolic dysfunction in T2D [171]. De Gonzalo-Calvo et al. demonstrated that exosomes isolated from lipid-loaded HL-1 cardiomyocytes had increased expression of cardiac-specific miRNA-1 and miRNA-133a compared to control cells [172]. They also identified robust increase in the expression of circulating miRNA-1 and − 133a in type 2 diabetic individuals [172], highlighting the value of circulating miRNAs as a diagnostic marker for developing diabetic cardiomyopathy [172]. However, the role of exosomal miRNA-1 and − 133a in circulation would need further study to determine the biomarker potential of exosomal miRNAs in diabetic cardiomyopathy.

Like other diseases, exosomes modified for their gene contents either through knockdown or overexpression have been used to treat of DCM. Induction of type 1 diabetes in transgenic mice overexpressed with HSP20 resulted in the production of beneficial exosomes compared to wild-type controls [99]. Notably, the study also showed that these exosomes, when transferred to the adjacent cells, induced beneficial effects, including angiogenesis, reduced oxidative stress, amelioration of fibrosis and apoptosis in the mouse diabetic heart [99]. Another study showed that cardiomyocyte-derived exosomes exhibited systemic effects and local effects. This study showed that both in vitro cellular stretch and in vivo pressure overload promote cardiomyocytes to produce exosomes enriched with angiotensin II type 1 receptor (AT1R [173]. These enriched exosomes were transferred to mesenteric vasculature and skeletal muscle to modulate peripheral vascular resistance and blood pressure [173].

The presented evidence shows that diabetes induces the release of exosomes from various cells, which plays a crucial role in the pathogenesis of the disease. On the other hand, treatments using exosomes derived from various types of stem cells or genetically modified exosomes with miRNA or genes showed therapeutic benefits in DHD. Under pathological conditions, exosomes released into circulation exhibit specific markers that can indicate the pathological state, making them a potential biomarker[23, 100]. In support of this, studies showed that exosomal miRNA expression was a better diagnostic marker for cardiac events in patients with coronary artery disease than freely circulating miRNAs [51]. This altered expression was primarily due to the selective sorting of miRNAs into exosomes in their host cells prior to release [51] (Pathophysiological role summarized in Fig. 2).

## Conclusion and future direction

This review summarised the current knowledge regarding the role of exosomes in CVD and DHD. Exosomes originating from cardiovascular cells provide a means for cell to cell communication by transferring miRNA, protein and cytokine cargos. Alterations in the exosomal cargo sorting in the host cells determine the physiological or pathological responses in the recipient cells. There are still some challenges that need to be overcome before its translation into routine clinical practice. For instance, multiple studies have demonstrated the potential therapeutic effects and biomarker capabilities of exosomal miRNAs. However, only a few have looked into the pathophysiological role of miRNA cargos in the DHD [23, 110, 174, 175]. While these studies provided evidence for the possible involvement of multiple miRNAs within the exosome cargos in the protective/destructive effects exhibited by the exosomes on their recipient cells [32, 175], they are only a fraction of the associated miRNAs with diabetes [8–10, 17, 176]. Additionally, most of these studies were either assessing individual disease conditions or had yet to delve into the complex molecular pathways affected by exosomal miRNA cargos [31, 33, 99, 110]. Therefore, further comprehensive analysis is essential in determining the full potential of exosomes as biomarkers and therapeutics for DHD.

Another caveat of the studies using exosomes is the lack of standardised isolation protocols for exosomes. This is primarily due to the requirement of a specific size range of exosomes. Due to the vast difference in the isolation procedures, different studies alternate between naming the vesicles as either exosome, extracellular vesicles or microvesicles. Although exosomes can be identified due to the expression of specific surface markers, studies have shown that these can be altered in response to diabetes or other associated comorbidities. Therefore, establishing a standardised protocol for isolating exosomes from tissues, cells and body fluids is crucial before translating these findings to the clinic.

## Data Availability

Not applicable.

## Abbreviations

AT1R: Angiotensin II type 1 receptor
CDC: Cardiosphere-derived cells
CPC: Cardiac progenitor cells
CVD: Cardiovascular disease
DCM: Diabetic cardiomyopathy
DHD: Diabetic heart disease
ECDE: Endothelial cells derived exosomes
EPC: Endothelial progenitor cells
ESCRT: Endosomal sorting complex required for transport
hESC-pg: human embryonic stem cell-derived cardiovascular progenitors
HSP: Heat shock protein
IHD: Ischemic heart disease
mbPDGF-BB: Membrane-bound platelet-derived growth factor-BB
MCEC: Mouse cardiac endothelial cell
MI: Myocardial infarction
miRNA: MicroRNA
MSC: Mesenchymal stem cells
MVB: Multivesicular bodies
PAD: Peripheral arterial disease
PYGB: Brain/head glycogen phosphorylase
RNA: Ribonucleic acid
SNARES: Soluble N-ethylmaleimide-sensitive fusion attachment protein receptors
T2D: Type 2 diabetes
VEGF: Vascular endothelial growth factor
VSMC: Vascular smoot muscle cells
